# Proteomic Analysis Reveals the Association between the Pathways of Glutathione and α-Linolenic Acid Metabolism and Lanthanum Accumulation in Tea Plants

**DOI:** 10.3390/molecules28031124

**Published:** 2023-01-23

**Authors:** Zhihui Xue, Zhidan Chen, Yankun Wang, Weijiang Sun

**Affiliations:** 1 College of Horticulture, Fujian Agriculture and Forestry University, Fuzhou 350002, China; 2 Anxi College of Tea Science, Fujian Agriculture and Forestry University, Quanzhou 362400, China; 3 China White Tea Research Institute, Fuding 355200, China; 4 Tea Industry Engineering Technology Research Center of Fujian Province, Fuzhou 350002, China; 5 Key Laboratory of Genetics, Breeding and Multiple Utilization of Crops, Ministry of Education, Fuzhou 350002, China

**Keywords:** rare earth elements, lanthanum, genotypes, Tieguanyin, Shuixian, *Camellia sinensis*

## Abstract

Lanthanum can affect the growth and development of the tea plant. Tieguanyin (TGY) and Shuixian (SX) cultivars of Camellia sinensis were selected to explore the mechanism underlying the accumulation of lanthanum (tea plants’ most accumulated rare earth element) through proteomics. Roots and fresh leaves of TGY and SX with low- and high-accumulation potential for lanthanum, respectively, were studied; 845 differentially expressed proteins (DEPs) were identified. Gene ontology analysis showed that DEPs were involved in redox processes and related to molecular functions. Kyoto Encyclopedia of Genes and Genomes metabolic pathway analysis showed that DEPs were associated with glutathione (GSH) and α-linolenic acid metabolism, plant pathogen interaction, and oxidative phosphorylation. Thirty-seven proteins in the GSH metabolism pathway showed significant differences, wherein 18 GSH S-transferases showed differential expression patterns in the root system. Compared with the control, expression ratios of GST (TEA004130.1) and GST (TEA032216.1) in TGY leaves were 6.84 and 4.06, respectively, after lanthanum treatment; these were significantly higher than those in SX leaves. The LOX2.1 (TEA011765.1) and LOX2.1 (TEA011776.1) expression ratios in the α-linolenic acid metabolic pathway were 2.44 and 6.43, respectively, in TGY roots, which were significantly higher than those in SX roots. The synthesis of specific substances induces lanthanum-associated defense responses in TGY, which is of great significance for plant yield stability.

## 1. Introduction

The tea plant (*Camellia sinensis* (L.) O. Kuntze) is a perennial woody plant. Fresh leaves are picked and processed to brew a healthy non-alcoholic beverage with a low calorific value. Tea is rich in a variety of nutritional and health-care components [1], such as tea catechins [2,3], L-theanine [4], and soluble sugar [5,6]. Rare earth elements (REEs) are non-essential elements that are present in organisms [7]. Spraying REEs on tea leaves can improve tea yield and quality [8,9], and they are mostly used as foliar fertilizers in many tea-producing areas [10]. However, the large-scale use of REEs inhibits the growth and development of plants [11]. REEs cause quality decline [12] and accumulate excessively in plants [13]. The REE content in roots was the highest, followed by the stem and leaves, and the REE content in leaves was positively correlated with maturity [14]. Analysis of REEs in tea plants showed that the content of lanthanum (La) was higher than that of heavy REEs [13,15]. 

Proteomics is the analysis of dynamic changes in protein composition, expression level, and modification state from an overall perspective. It can help to understand the interaction and connection between proteins and reveal rules of protein function and cell life activities. Proteins are the product of gene expression and are executors of life activities. Therefore, studying biological processes at the protein level has important physiological significance for understanding the laws of biological activities. The mechanism of plant resistance to stress caused by heavy metals has been analyzed through proteomics, with some progress [16,17,18].

We determined the REE content in 55 tea plants with one bud and three leaves and screened out the low- and high-REE-absorbing experimental cultivars of *C. sinensis,* namely, Tieguanyin (TGY) and Shuixian (SX), respectively [19]. Further study showed that the La absorption in the leaves of TGY was lower than that in the leaves of SX at an La concentration of 1.20 mmol/L, and the La accumulation in the roots of SX was 4.61 times that in the roots of TGY. Scanning electron microscopy and transmission electron microscopy showed that the subcellular structures of the roots and leaves of TGY and SX were different [20,21]. However, the molecular mechanisms of La accumulation in TGY and SX have rarely been reported. Therefore, in this study, the leaves and roots of TGY and SX were used to study their differentially expressed proteins (DEPs) in response to La treatment using proteomic technology. Furthermore, metabolic pathways of differences in La accumulation in tea plants were revealed. These observations provide a reference for further clarification of the molecular mechanism underlying the reduced accumulation of REEs in tea plants.

## 2. Results

### 2.1. Effect of La on Tea Growth and La Accumulation

**Figure 1:**
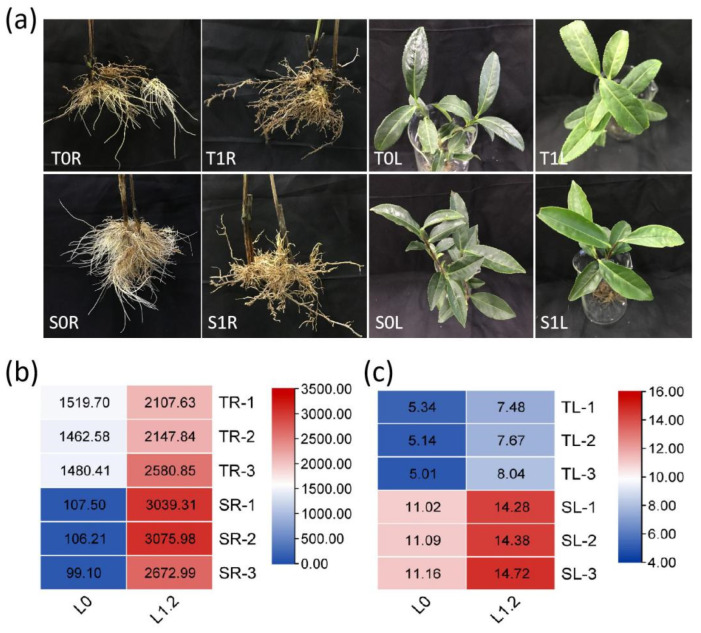
Effect of La on tea growth and La accumulation. (**a**) Growth of Shuixian and Tieguanyin under La treatment; (**b**) La accumulation in the roots of Shuixian and Tieguanyin, Units: mg/kg; (**c**) La accumulation in the leaf of Shuixian and Tieguanyin, Units: mg/kg. T0L, Tieguanyin leaf under the control treatment; T1L, Tieguanyin leaf treated with 1.2 mmol/L La; S0L, Shuixian leaf under the control treatment; S1L, Shuixian leaf treated with 1.2 mmol/L La; T0R, Tieguanyin roots under the control treatment; T1R, Tieguanyin roots treated with 1.2 mmol/L La; S0R, Shuixian roots under the control treatment; S1R, Shuixian roots treated with 1.2 mmol/L La.

Under the control treatment, the two tea varieties were flourishing—the new shoots were mostly normal mature new shoots and both varieties produced a large number of new roots. After treatment with 1.20 mmol/L La, leaf growth declined, leaf color became yellowish-green, and luster was lost; moreover, the growth and development of new shoots were delayed, the root system was yellowish-brown and became coarse when old, and no new roots were produced (Figure 1a).

Compared with the control, the La content in the root and leaf parts of TGY and SX increased after 1.20 mmol/L La treatment, and the La content in the root was significantly higher than that in the leaf. Further, the La content in the root and leaf of TGY varieties was lower than those of SX varieties (Figure 1b,c).

### 2.2. Qualitative Assessment of Protein Assay Data

**Figure 2:**
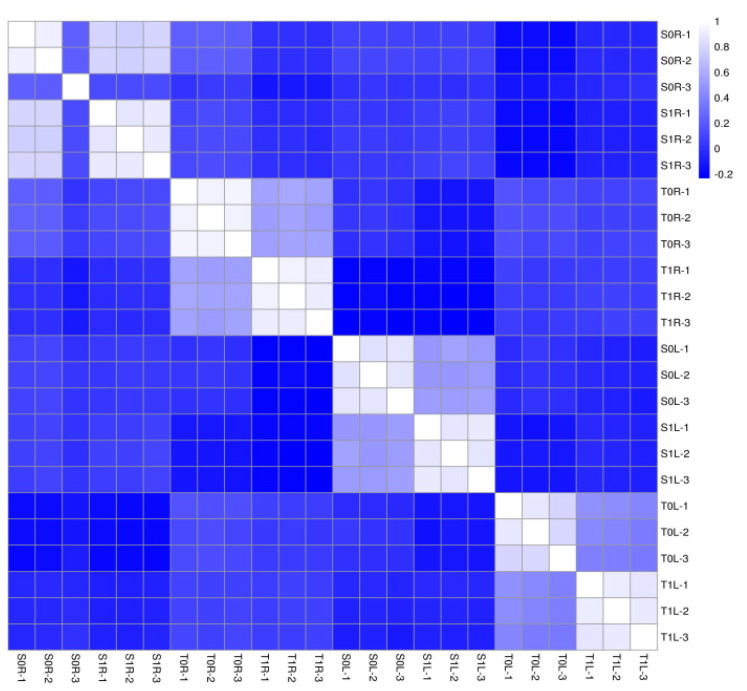
Heat map of correlation coefficient between samples.

The regression equation of the standard curve was y = 0.1809x + 0.4596, with a correlation coefficient R^2^ of 0.9961. The correlation coefficient between the protein expression levels of each sample was >0.84 (Figure 2), indicating that protein data of the tested samples showed good repeatability. 

### 2.3. Differential Protein Analysis of the Roots and Leaves of TGY and SX under La Treatment

**Figure 3:**
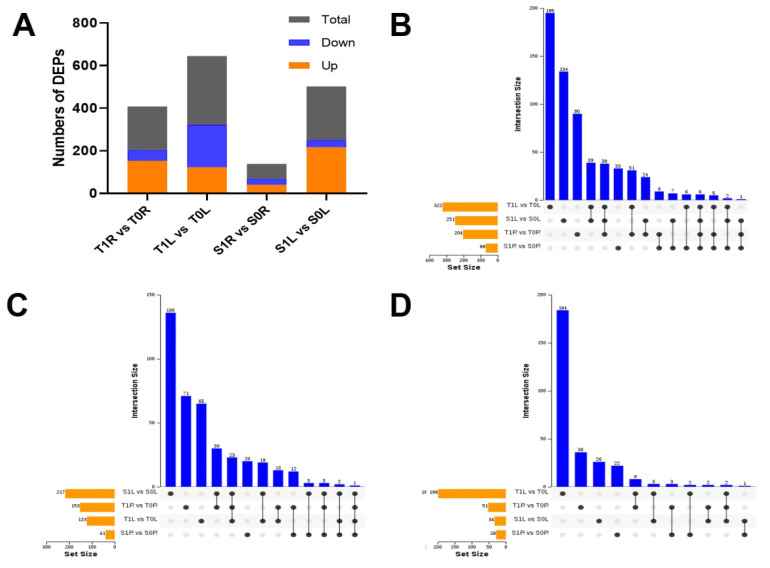
Analysis of DEPs in the roots and leaves of Tieguanyin and Shuixian under La treatment and UpSet Venn diagram. (**A**) DEP numbers in roots and leaves of Tieguanyin and Shuixian under La treatment. (**B**) UpSet Venn diagram of DEPs in the compared group. (**C**) UpSet Venn diagram of upregulated expressed proteins in the compared group. (**D**) UpSet Venn diagram of downregulated expressed proteins in the compared group. T0L, Tieguanyin leaf under the control treatment; T1L, Tieguanyin leaf treated with 1.2 mmol/L La; S0L, Shuixian leaf under the control treatment; S1L, Shuixian leaf treated with 1.2 mmol/L La; T0R, Tieguanyin roots under the control treatment; T1R, Tieguanyin roots treated with 1.2 mmol/L La; S0R, Shuixian roots under the control treatment; S1R, Shuixian roots treated with 1.2 mmol/L La.

To understand the effect of La on tea plants, root and leaf proteome profiles were obtained using a label-free proteomics approach. There were more upregulated proteins in T1R than in T0R (up 153 > down 51) and more upregulated proteins in S1R than in S0R (up 41 > down 28), indicating that different root proteins of TGY and SX were upregulated under La treatment. In leaves, there were more downregulated proteins in T1L than in T0L (down 199 > up 123) and more upregulated proteins in S1L than in S0L (up 217 > down 34) (Figure 3A), indicating that under La treatment, the differential proteins in the leaves of TGY and SX showed different expression patterns. The DEPs in leaves of TGY were downregulated, whereas those in the leaves of SX were upregulated.

UpSet Venn diagram analysis of DEPs in the roots and leaves of TGY and SX under La treatment showed that only 195, 90, and 134 DEPs were found in the leaves of TGY, roots of TGY, and leaves of SX, respectively. There were 33 DEPs in the roots of SX (Figure 3B), and these proteins may be specific to different parts of TGY and SX that respond to La treatment. The numbers of DEPs in the roots and leaves of TGY were the highest, indicating that La treatment can induce the production of more specific proteins in TGY. Compared with the control, there were 19 upregulated and three downregulated DEPs shared by the leaves of TGY and SX and 12 upregulated and three downregulated DEPs shared by the roots of TGY and SX (Figure 3C,D). The roots of TGY and SX produce a large number of commonly expressed proteins under La treatment, and the number of upregulated DEPs is greater than that of downregulated DEPs.

### 2.4. Bioinformatic Analysis of DEPs in the Roots and Leaves of TGY and SX under La Treatment

Gene ontology (GO) analysis revealed that the DEPs in tea roots and leaves were involved in several biological processes, as shown in Figure S1, see Supplymentary Materials. In the roots and leaves of TGY, DEPs participated in metabolic processes, cellular processes, biological regulation, and response to stimuli (Figure S1A,B). In the roots and leaves of SX, metabolic processes, single biological processes, and cellular processes occupied the top three biological processes (Figure S1C,D). DEPs in the roots and leaves of TGY were distributed in cells, cell components, organelles, membranes, and macromolecular complexes (Figure S1A,B), whereas DEPs in the roots and leaves of SX were distributed in cells, cell components, and organelles (Figure S1C,D). For molecular function, DEPs in the roots and leaves of TGY were involved in catalytic, binding, transport, and antioxidant activities, and the regulation of molecular function (Figure S1A,B). DEPs in the roots and leaves of SX were highly enriched in catalytic, binding, and antioxidant activities (Figure S1C,D).

Kyoto Encyclopedia of Genes and Genomes (KEGG) enrichment analysis showed that DEPs in roots and leaves of tea plants were involved in several pathways (Figure S2). The DEPs from roots and leaves of TGY were enriched in phenylpropane biosynthesis and galactose, α-linolenic acid, and phenylalanine metabolisms (Figure S2A,B). The DEPs in roots and leaves of SX were enriched in α-linolenic acid and glutathione (GSH) metabolisms, monoterpenoid biosynthesis, and plant-hormone signal transduction (Figure S2C,D). In addition to the enriched pathways, some important metabolic pathways in response to La were also observed in roots and leaves of TGY and SX, including biosynthesis of flavonoids and amino acids, plant–pathogen interaction, and the phosphatidylinositol signaling system.

### 2.5. Analysis of the Expression of Key Proteins in the GSH Metabolic Pathway in Different TGY and SX Sites in Response to La

Based on the differential expression and enrichment results of proteins of TGY and SX in different organs, the key proteins in the GSH metabolic pathway related to environmental stress were selected. A metabolic pathway heat map was drawn to further analyze the pattern of response of protein levels of TGY and SX to La treatment in different tissues. 

**Figure 4:**
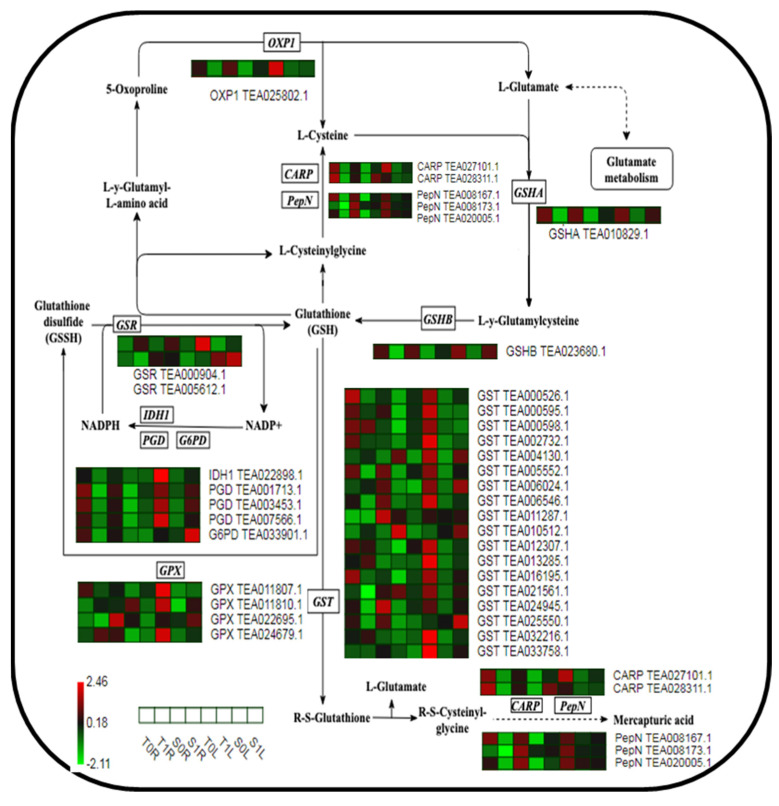
Differential protein expression patterns in the GSH metabolism pathway in roots and leaves of Tieguanyin and Shuixian under La treatment. T0L, Tieguanyin leaf under the control; T1L, Tieguanyin leaf treated with 1.2 mmol/L La; S0L, Shuixian leaf under the control; S1L, Shuixian leaf treated with 1.2 mmol/L La; T0R, Tieguanyin roots under the control treatment; T1R, Tieguanyin roots treated with 1.2 mmol/L La; S0R, Shuixian roots under the control; S1R, Shuixian roots treated with 1.2 mmol/L La.

As an antioxidant, GSH plays an important role in resisting the harmful effects of heavy metals. GSH can remove reactive oxygen species (ROS) through enzyme or non-enzyme systems and can also alleviate toxicity by catalyzing the binding of GSH to heavy metal ions through GSH S-transferase (GST) [22]. In the biosynthesis of GSH, GSH synthases (GSHA; TEA010829.1 and TEA023680.1), which catalyze the synthesis of GSH from glutamic acid and cysteine, had higher expression levels in T1L and S1L. However, the expression levels of T1R and S1R were downregulated, which promoted GSH synthesis in the leaves of TGY and SX and decreased GSH synthesis in their roots. GSH was decomposed into L-cysteinylglycine under the action of glutamyl transpeptidase and then into cysteine under the action of the leucyl aminopeptidase CARP and aminopeptidase PepN, which were upregulated in T1L and significantly downregulated in T1R and S1R, reducing GSH decomposition (Figure 4). Glutathione peroxidase (GPx), which catalyzes the conversion of GSH to oxidized GSH (GSSH), was upregulated in both T1L and S1L, and the upregulated level of T1L was significantly higher than that of S1L. The expression level of T1R was also higher than that of S1R, which promoted GSH oxidation in the roots and leaves of TGY and slowed down ROS production by TGY stimulated by La. Concurrently, PGD, IDH1, and G6PD, which catalyze the reduction of NADP+ to NADPH, were upregulated in T1L and S1L. The upregulated level of T1L was significantly higher than that of S1L but was downregulated in T1R and S1R. The reduction of oxidized GSH (GSSG) to GSH was catalyzed by the GSH reductase protein GSR to maintain the dynamic equilibrium of GSH.

GST is a GSH-dependent ROS detoxification enzyme that exists in the plant cytoplasm, and it can catalyze the binding of GSH to various hydrophobic and electrophilic organic molecules to produce water-soluble, inactive, and low-toxic or non-toxic peptide derivatives. In the GSH metabolic pathway, a total of 37 proteins were significantly changed, and GST accounted for the majority (18 in total). Compared to that in the control, all GSTs were significantly upregulated in T1L and S1L, but the upregulated level in T1L was significantly higher than that in S1L. The expression multiples of GST (TEA004130.1) and GST (TEA032216.1) in T1L vs. T0L were 6.84 and 4.06, respectively, while those in S1L vs. S0L were 5.87 and 2.48, respectively. However, GST showed different expression patterns in the roots. Eleven GST enzymes were downregulated in T1R and S1R, and five were upregulated in T1R and S1R; in particular, GST (TEA006024.1) and GST (TEA011287.1) were upregulated in T1R vs. T0R. The downregulated expression levels of S1R and S0R affect different metabolic responses of TGY and SX in their root and leaf tissues and regulate the accumulation of La in TGY and SX.

### 2.6. Analysis of the Expression of Key Proteins in Theα-Linolenic Acid Metabolism Pathway in Different TGY and SX Sites in Response to La

**Figure 5:**
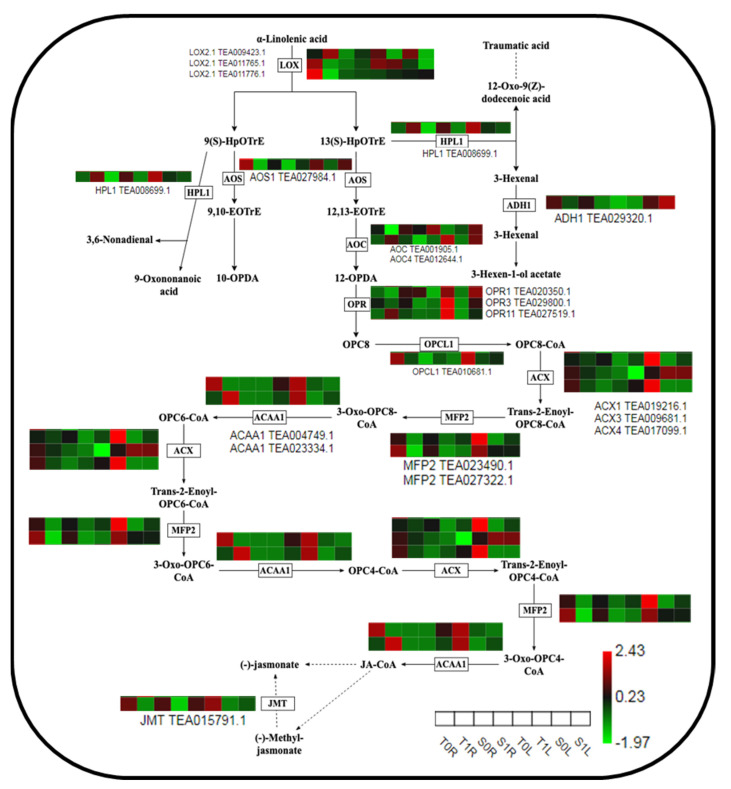
Differential protein expression pattern in the α-linolenic acid metabolism pathway in the roots and leaves of Tieguanyin and Shuixian under La treatment. T0L, Tieguanyin leaf under the control treatment; T1L, Tieguanyin leaf treated with 1.2 mmol/L La; S0L, Shuixian leaf under the control treatment; S1L, Shuixian leaf treated with 1.2 mmol/L La; T0R, Tieguanyin roots under the control treatment; T1R, Tieguanyin roots treated with 1.2 mmol/L La; S0R, Shuixian roots under the control treatment; S1R, Shuixian roots treated with 1.2 mmol/L La.

The α-linolenic acid metabolic pathway is an important signaling pathway in plant-induced defense. The expression levels of 20 proteins involved in the α-linolenic acid metabolic pathway were altered by La treatment, including lipoxygenase (LOX), propylene synthase (AOS), ethanol dehydrogenase (ADH1), propylene cyclase (AOC), oxyphytate reductase (OPR), coumaric acid coenzyme A ligase (OPCL), acetyl-coenzyme A oxidase (ACX), enoyl-coenzyme A hydrase (MFP2), ketone lipids, acyl-coenzyme A thiolase (ACAA), jasmonate methyl transferase (JMT), and other enzymes. The α-linolenic acid catabolism rate-limiting enzyme LOX was downregulated in La-treated leaves but downregulated and upregulated in T1R and S1R, respectively. The expression multiples of LOX2.1 (TEA011765.1) and LOX2.1 (TEA011776.1) in T1R vs. T0R were 2.44 and 6.43, respectively, while those of S1R vs. S0R were 1.98 and 1.09, respectively. The key downstream enzymes AOS, AOC, OPR, OPCL, ACX, MFP2, and ACAA of the third β-oxidation of fatty acids and JMT protein-catalyzed jasmonic acid (JA) methylation were significantly downregulated in T1R and S1R but significantly upregulated in T1L and S1L. Moreover, the expression level in T1L was higher than that in S1L, which promoted the synthesis of JA in T1L, provided the material basis for the JA signaling pathway, and further induced the defense regulation of leaves of TGY against La (Figure 5).

## 3. Discussion

### 3.1. Analysis of Differential Protein Enrichment in the Roots and Leaves of TGY and SX Treated with La

In this study, 204, 322, 69, and 251 DEPs were identified in T1R vs T0R, T1L vs T0L, S1R vs S0R, and S1L vs S0L, respectively. Under La treatment, differential proteins of roots of TGY and SX were regulated. The DEPs in the leaves of TGY and SX showed different expression patterns. DEPs in the leaves of TGY were downregulated, whereas those in the leaves of SX were upregulated. GO enrichment analysis showed that the DEPs of TGY were enriched in the redox process, defense reaction, oxidative stress reaction, catalytic activity, and metal ion-binding processes, while those of SX were enriched in metabolic processes, catalytic activity, and organelles. KEGG metabolic pathway analysis indicated that the DEPs of TGY were enriched in alpha linolenic acid, GSH, and phenylalanine metabolisms; plant–pathogen interactions; oxidative phosphorylation; and flavonoid biosynthesis, while those of SX were enriched in the biosynthesis of benzene propane, flavonoid, and single terpenoid and metabolic pathways of phenylalanine, starch, and sucrose. This is similar to the differential protein enrichment pathways in response to cadmium stress in cadmium-resistant and cadmium-sensitive rice varieties studied by Lin et al. [23] and the enrichment pathway in response to copper stress in rice leaves studied by Hajduch et al. [24]. Abnormal expression of antioxidant defensive proteins occurs during photosynthesis, thereby improving plant resistance. We previously conducted transcriptome sequencing on T15, Benshan, and Shuixian from three tea germplasms with different REE accumulation levels, and the enrichment pathways of differentially expressed genes were plant-hormone signal transduction metabolic pathways and glutathione metabolic pathways, which was consistent with the results of a previous study [25]. These results indicate that a series of metabolic pathway changes occurred in the roots and leaves of TGY and SX under La treatment, and the differences in gene and protein expression patterns caused the differences in La accumulation between the two varieties.

### 3.2. Different Parts of TGY and SX Respond to La via the Protein Regulation Mechanism of the GSH Metabolic Pathway

Endogenous plant GSH, its redox state (GSH/GSSG), and GSH metabolism-related enzymes (glutathione reductase, GPx, and GST) play a central role in the generation and maintenance of redox signals by ROS under detoxification conditions [26]. GSHA and GSHB, the key enzymes of GSH biosynthesis, were upregulated in T1L and S1L but downregulated in T1R and S1R, indicating that La treatment promoted the synthesis of GSH in the leaves of TGY and SX. However, because the root system was the main absorption site, it was more strongly affected by La, which resulted in the downregulation of this kind of protein in the root system. GST is a polygenic isoenzyme that catalyzes the binding of GSH to toxic compounds and alleviates oxidative stress by scavenging ROS [27,28]. GPx plays an important role in regulating the GSH/GSSG ratio to maintain a high reduction capacity and transmit ROS signals to the redox signal network [29]. In this study, the key enzymes, GPx and GST, which catalyzed the oxidative degradation of GSH, PGD, IDH1, and G6PD, and the reduction of NADP+ to NADPH, were all upregulated in T1L and S1L, and the upregulation level of T1L was significantly higher than that of S1L. This indicates that the leaves of TGY under La treatment can better promote the oxidation of GSH and the combination with electrophilic substances than the leaves of SX, effectively alleviating the damage caused by ROS to the leaves of TGY and improving the La resistance of TGY. This is consistent with the growth and development of the leaves of TGY under La treatment compared with SX [21]. In addition, the observation of cell ultrastructure showed that the degree of damage of TGY leaf cells was lower than that of SX leaf cells, which was consistent with the results of a study by Roxas et al. [30], which stated that overexpression of GST and GPx activity in transgenic tobacco increased the clearance of GSH-dependent peroxide and the increase in GSH content, leading to less oxidative damage.

However, the expression of these key proteases in the roots of TGY and SX was different from that in the leaves, especially that of a large number of GST proteases, of which two GSTs, TEA006024.1 and TEA011287.1, were upregulated in T1R vs. T0R, but their expression in S1R vs. S0R was downregulated. This indicates that, after La treatment, GST protease in the roots of TGY was significantly upregulated, catalyzed GSH, caused excessive La accumulation, and eliminated ROS to reduce the damage by La to TGY, resulting in TGY having a strong tolerance and low La accumulations, which is consistent with our previous physiological studies. We found that after La treatment, the antioxidant enzyme activity of the TGY roots with a low accumulation increased, the ultrastructure of the root cells suffered less damage, and the accumulation of La was lower [21]. These results further confirmed that the GSH-related antioxidant defense system induced by La may be the main mechanism underlying the difference in La accumulation between TGY and SX. These results also provide a theoretical basis for further analysis of the mechanism by which GSH regulates the difference between TGY and SX in REE accumulation.

### 3.3. Different Parts of TGY and SX Respond to La via the Protein Regulation Mechanism of the α-Linolenic Acid Metabolic Pathway

The octadecane pathways starting from linolenic and hexadecenoic acids are pathways for JA synthesis. JA compounds play an important role in plant responses to abiotic stress and regulate plant defense processes. The enzymes involved in the initiation of JA synthesis from linolenic acid are not only necessary for JA synthesis but are also important signaling molecules involved in plant-induced defense. In this study, AOS, AOC, OPR, OPCL, ACX, MFP2, and ACAA, key enzymes of the third β-oxidation of fatty acids and JMT that catalyzes JA methylation, showed a significant upregulated expression pattern in T1L vs. T0L and S1L vs. S0L, and the expression level in T1L was higher than that in S1L. These results indicate that the leaves of TGY promote JA synthesis more than *Narcissus* leaves under La treatment, which is then released into the cytoplasm to bind to free receptors, initiating the next reaction, inducing the defense regulation of the leaves of TGY on La, and improving the tolerance of TGY to La. As a result, TGY showed vigorous growth, and an improvement in both yield and quality. However, these key regulatory enzymes showed different induction patterns in the roots of the two cultivars compared with those of leaves and were significantly downregulated in T1R vs. T0R and S1R vs. S0R. Moreover, their upregulation was inconsistent between T1R and T0R, suggesting the presence of post-transcriptional and post-translational modifications, protein folding, stability and localization, and protein–protein interactions of these key genes in the α-linolenic acid metabolism pathway of the roots of TGY.

## 4. Materials

### 4.1. Plant Materials

One-year-old root cuttings of the low- and high-REE-absorbing experimental cultivars of *C. sinensis*, TGY and SX, respectively, were selected as plant materials. Cuttings were obtained from a local commercial tea seedling farm (Anxi Qianhe). Each plant, regarded as a seedling, was 20–30 cm long with a main stem diameter of 2–3 mm. Plants of both cultivars showed similar growth rates.

### 4.2. Hydroponic Culture of Tea Seedlings

**Figure 6:**
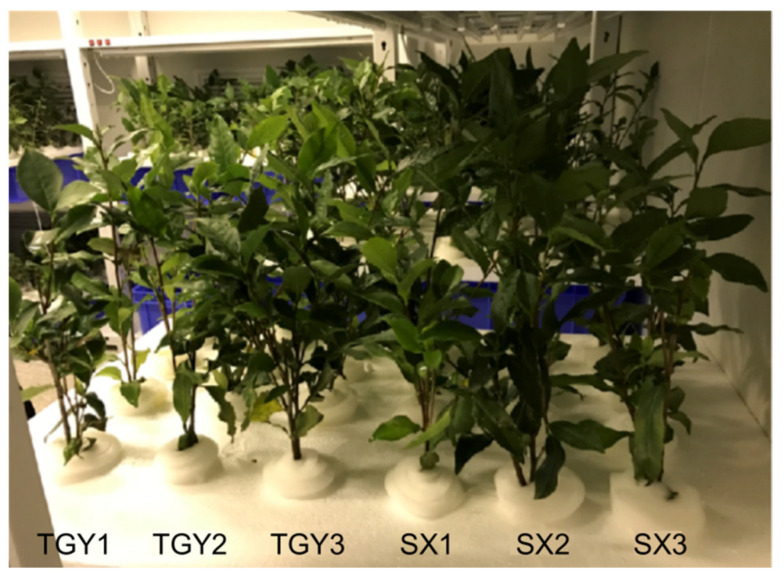
Experimental setup of tea seedlings in varying nutrient solutions for culture used in this study.

The experimental tea seedlings were washed under tap water, and their roots were rinsed with deionized water. Cuttings of TGY and SX were cultured in a growth chamber in containers with a capacity of 35 L and a height of 15 cm (Turnover Box Co., Nanjing, China) with 4–6 holes on the top. Individual plants were separated using a foam board, and a sponge was tightly inserted into each hole for the stems to protrude through. Roots were shielded from light, and a half of the nutrient solution was replaced at weekly intervals (Figure 6). The compositions of nutrient solutions are listed in Table 1. The pH of the solution was maintained between 5.0 and 5.5. Tea seedlings were cultured in this system for 2–3 weeks prior to the initiation of treatment. The experiment was conducted in an artificial climate chamber under a day/night temperature regime of 28/20 °C and relative humidity of 75%. Seedlings of both varieties grew uniformly before the initiation of treatment. To observe the growth of a large number of new white roots compared to the original roots, we added La^3+^ at 1.20 mmol/L.

### 4.3. La Treatment

Analytical-grade La chloride (LaCl_3_·6H_2_O) was used to prepare different concentrations of La^3+^ solutions. In this study, the concentration of La^3+^ was set at 1.20 mmol/L and distilled water was set as the control. Solutions were prepared by adding an appropriate amount of LaCl_3_·6H_2_O to distilled water. After heating, the solutions were cooled to 25 °C and quantified to a final volume of 1 L. Each treatment was experimentally performed in triplicate, with 48 plants per replicate, to ensure an adequate number of robust plants to measure results.

### 4.4. Determination of La in Roots and Leaves

Having exposed hydroponic-cultured tea seedlings to La treatments for 30 days, the seedlings were washed with ultra-pure water, and the newly developed roots and leaves were sampled from approximately 10 plants per treatment. The collected samples were oven-dried at 103 °C. After grinding, samples were sifted through a 40-mesh sieve, then packaged in self-sealing bags, labeled, and stored until analysis. 

Samples were prepared for quantitative analysis using microwave digestion. Briefly, 0.2 g samples were weighed and placed in a high-pressure digestion tank, to which 7.5 mL of HNO_3_ and 1.5 mL H_2_O_2_ were added. The samples were then left to stand for 1 h, prior to digestion in a microwave digestion device. After digestion and cooling, the air was slowly exhausted, and the acid was heated to 140 °C on a heating plate, following which the digested samples were transferred to 50 mL volumetric flasks, and the digestion tank was washed three times with a small amount of deionized water. Aliquots (8 mL) of the solution obtained from each digested sample were then filtered using a 0.22 μm water system filter-head and placed in 10 mL centrifuge tubes. A blank tube containing deionized water was used as a control. The determination of REEs was conducted using inductively coupled plasma mass spectrometry (iCAP Q; Thermo Fisher Scientific™, Waltham, MA, USA) with reference to GB 5009.94-2012 “National Food Safety Standard for Determination of Rare Earth Elements in plant foods”. 

### 4.5. Identification and Analysis of Protein Using Mass Spectrometry

#### 4.5.1. Protein Extraction and Trypsin Digestion 

Roots of TGY and SX (T0R/T1R/S0R/S1R) and the third leaf of the shoots (T0L/T1L/S0L/S1L) were frozen in liquid nitrogen at concentrations of 0 and 1.2 mmol/L, respectively. Protein samples were extracted using the Total Plant Protein Extraction Kit (PP0601-10, Beijing Bonfei Biotechnology Co., Ltd., Beijing, China), and protein quality inspection was conducted using the Bradford Protein Quantitative Kit (PA121221, Tiangen Biotech (Beijing) Co., Ltd, Beijing, China). Protein samples (35 µg) were taken from each sample, and 5× sample loading buffer was added at a ratio of 5:1 (*v*/*v*) and bathed in boiling water for 5 min; then, at 14,000× *g*, the supernatant was centrifuged for 10 min and subjected to 10% sodium dodecyl sulfate-polyacrylamide gel electrophoresis (SDS-PAGE) (constant flow: 14 mA; electrophoresis time: 90 min). After electrophoresis, sample cleavage was evaluated using Coomassie brilliant-blue staining.

For digestion, the protein solution was reduced with 5 µL 1M DTT for 60 min at 37 °C and alkylated with 200 µL 1M IAA for 60 min at 26 °C in darkness. Next, the detergent, DTT, and other low-molecular-weight components were removed using UA buffer (8M Urea) by repeated ultrafiltration (Sartorius). The filters were washed with 100 μL UA buffer twice and then with 100 μL 50 mM NH_4_HCO_3_ buffer three times. Each sample was digested at a ratio of 1:50 (trypsin: protein concentration) at 37 ℃ overnight, and the resulting peptides were collected as a filtrate. The peptide of each sample was desalted on C18 Cartridges, and then concentrated by vacuum centrifugation and reconstituted in 40 µL 0.1% (*v*/*v*) formic acid. The peptide content was estimated by UV light spectral density at 280 nm using an extinction coefficient of 1.1 for the 0.1% (g/L) solution, which was calculated on the basis of the frequency of tryptophan and tyrosine in vertebrate proteins.

#### 4.5.2. Peptide Identification by LC-MS/MS

LC-MS/MS analysis was performed on Q Exactive HF mass spectrometer (Thermo Fisher Scientific) that was coupled to HPLC (Thermo Fisher Scientific). Peptide was loaded onto the C18-reversed phase analytical column in buffer A (0.1% formic acid) and separated with a linear gradient of buffer B (80% acetonitrile and 0.08% Formic acid) at a flow rate of 600 nL/min. The linear gradient is shown in Table 2.

MS data were acquired using a data-dependent top 10 method for dynamically choosing the most abundant precursor ions from the survey scan (350–1400 *m*/*z*) for HCD fragmentation. MS1 scans were acquired at a resolution of 120,000 with an AGC target of 3e6 and a maxIT of 80 ms. MS2 scans were acquired at a resolution of 15,000 at 120 *m*/*z* with an AGC target of 5e4 and a maxIT of 19 ms, and isolation width was 1.6 *m*/*z*. Normalized collision energy was 27 eV.

#### 4.5.3. Proteome Data and Bioinformatics Analysis

All proteomic samples were designed with three biological replicates. For analysis of protein, the tea genome (*C. sinensis* var assamica) database was employed [31], and the data were searched and quantified using Proteome discoverer software 2.4 (Thermo Fisher Scientific Inc., Waltham, MA, USA).

According to the *p*-value of the primary data, data with *p* ≤ 0.05 and fold change ≥ 1.2 were selected for further analysis. Functional annotation and enrichment analysis were performed using GO (https://www.geneontology.org/ (accessed on 30 October 2022)), and metabolic pathway analysis of proteins was performed using KEGG (https://www.genome.jp/kegg (accessed on 30 October 2022)).

## Figures and Tables

**Table 1:** Composition of the hydroponic solution used for the culture of tea seedlings.

Element	Source	Concentration mmol/L
Al	Al_2_(SO_4_)_3_·18H_2_O	0.25
NH_4_^−^N	(NH_4_)_2_SO_4_	0.75
NO_3_^−^N	NH_4_NO_3_	0.25
P	KH_2_PO_4_	0.05
K	K_2_SO_4_	0.35
Ca	CaCl_2_	0.395
Mg	MgSO_4_	0.21
B	H_3_BO_3_	3.33
Mn	MnSO_4_·H_2_O	0.5
Zn	ZnSO_4_·7H_2_O	0.51
Cu	CuSO_4_·5H_2_O	0.13
Mo	Na_2_MoO_4_·2H_2_O	0.17
Fe	Fe-EDTA	2.10

**Table 2:** Liquid chromatography elution gradient parameters.

Time (min)	A (0.1% FA, H_2_O)	B (0.08% FA, 80% ACN)	Flow Rate (nL/min)
0	95%	5%	600
16	90%	10%	600
51	78%	22%	600
71	70%	30%	600
72	5%	95%	600
78	5%	95%	600

## Data Availability

The raw sequence data were deposited in the Proteomics Identification Database, PRIDE, under accession number PXD039195, which is publicly accessible at https://www.ebi.ac.uk/pride/, accessed on 3 January 2023.

## Supplementary Materials

The following supporting information can be downloaded at: https://www.mdpi.com/article/10.3390/molecules28031124/s1.
