# Supplementary figures and images for: Proteomic Analysis Reveals the Association between the Pathways of Glutathione and α-Linolenic Acid Metabolism and Lanthanum Accumulation in Tea Plants
Source: Molecules. 2023 Jan 23;28(3):1124. doi: 10.3390/molecules28031124 (PMC9920552; doi:10.3390/molecules28031124)

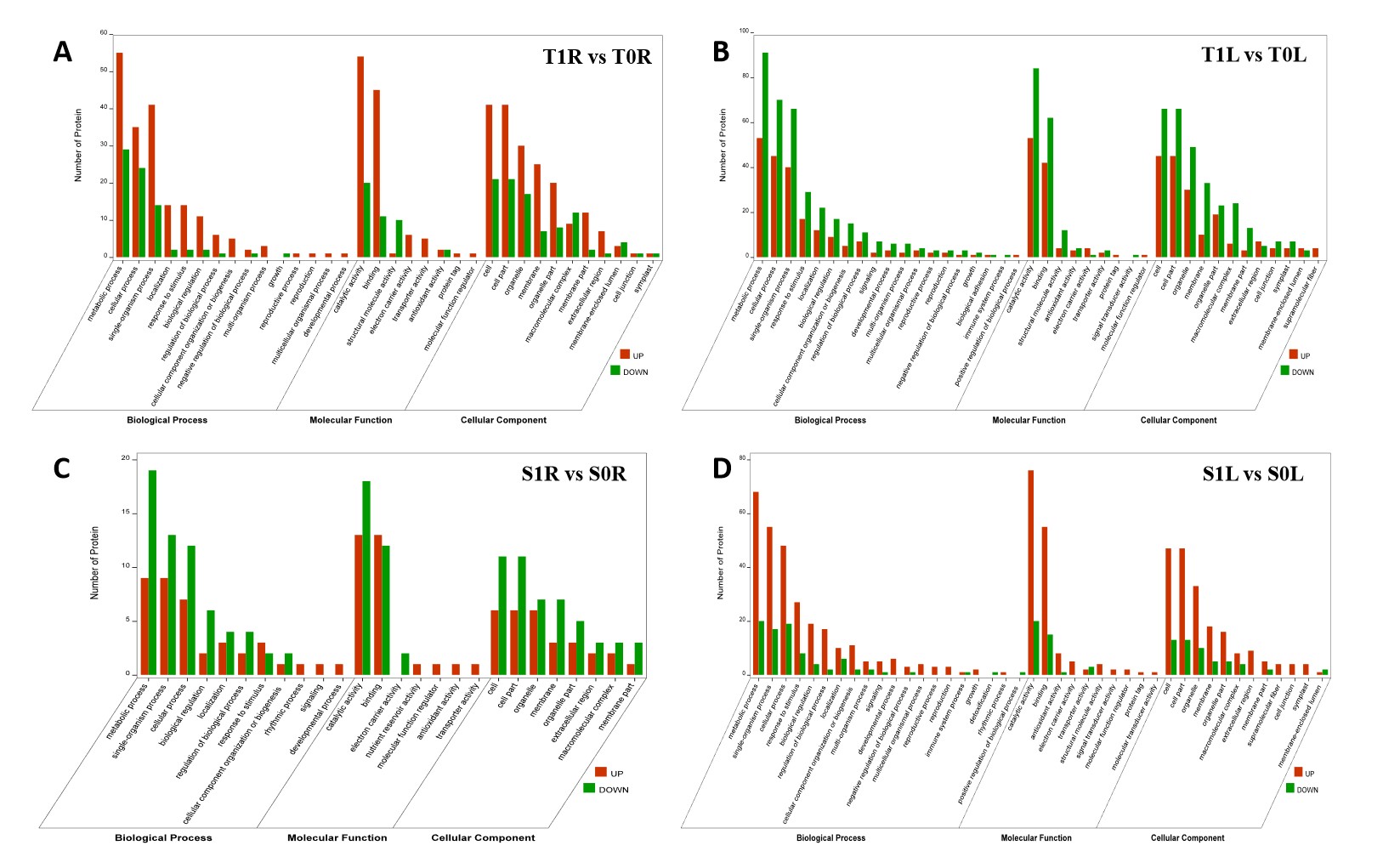

Supplement: Supplementary file 1 [file molecules-28-01124-s001.zip › Figure S1.jpg]

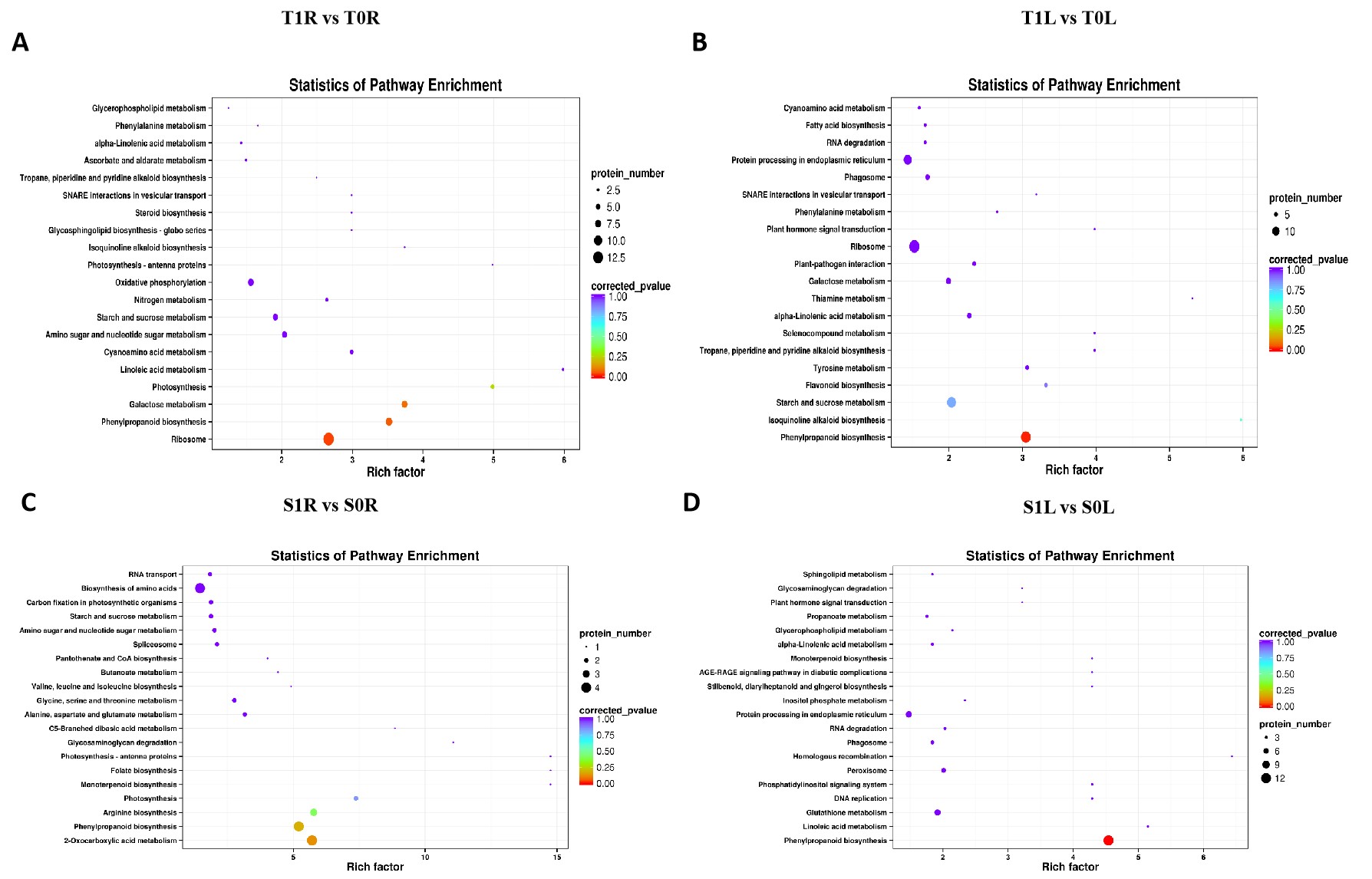

Supplement: Supplementary file 1 [file molecules-28-01124-s001.zip › Figure S2.jpg]
